# Seasonal dynamics of seed dormancy and germination in the weed *Diplachne fusca*

**DOI:** 10.7717/peerj.17987

**Published:** 2024-08-30

**Authors:** Guiquan Song, Shufang Liu, Xuelian Jiang, Shuai Gong, Wenya Hao, Ying Cui, Yueling Zhao

**Affiliations:** 1 Weifang University, Weifang, China; 2 Sinochem Agriculture Holdings Co. Ltd, Beijing, China

**Keywords:** *Diplachne fusca*, Dormancy break, Dormancy/non-dormancy cycle, Light, Seed germination, Weed

## Abstract

**Background:**

Understanding the reproductive biology of weeds is crucial for managing them effectively. *Diplachne fusca* (Poaceae) is a widely distributed weed species that poses significant challenges to agricultural productivity. Nevertheless, it remains unclear how the soil seed bank of *D. fusca* responds to environmental shifts, and whether a dormancy cycle is present in this species.

**Methods:**

We investigated how seed dormancy in *D. fusca* is broken and how it responds to natural environmental changes. The impact of incubation temperature, light exposure, cold stratification at 4 °C, and gibberellic acid (GA_3_) on seed germination/dormancy-break was investigated, along with assessing seasonal changes in germinability through monthly excavation and laboratory incubation of buried seeds over 2 years.

**Results:**

Results indicated that newly ripened seeds of *D. fusca* were dormant, with germination facilitated by GA_3_, cold stratification, and after-ripening at ambient room conditions. Exposure to darkness inhibited germination. Seasonal patterns of germination were observed, with peak germination occurring in cooler months and a marked decline during the hot summer months. After 2 years of being buried, approximately 40% of the seeds remained viable.

**Conclusion:**

In summary, seeds of *D. fusca* exhibit non-deep physiological dormancy and maintain a persistent soil seed bank. Seeds buried in the soil undergo a yearly dormancy/non-dormancy cycle. This dormancy cycle prevents seed germination and seedling emergence in autumn, which boosts the survival of seedlings in less favorable seasons, yet it also makes it more challenging to eradicate this weed.

## Introduction

Weeds present numerous challenges in both agricultural and natural environments (Yuan et al., 2019; Driver, Al-Khatib & Godar, 2020a; Maity et al., 2023; Umurzokov et al., 2024). They significantly reduce crop yields and degrade the quality of harvested produce by fiercely competing for light, water, and nutrients (Umurzokov et al., 2024). Additionally, dense weed infestations complicate mechanical harvesting, increasing labor costs and disrupting essential farming operations (Holm et al., 1997), while also serving as reservoirs for pests and diseases that can spread to and devastate neighboring crops (Umurzokov et al., 2024). Managing these invasions demands substantial financial resources, thereby adding economic strain to farming operations. Furthermore, in natural ecosystems, invasive weeds threaten biodiversity by outcompeting native species, disrupting habitats, and contributing to long-term environmental degradation (Umurzokov et al., 2024). Effective weed management is therefore essential for improving agricultural productivity.

Understanding the reproductive biology of weeds is crucial for managing them effectively. Firstly, many weeds propagate through seeds and are prolific producers of seeds (Netto et al., 2022). For instance, *Panicum capillare* can produce up to 56,400 seeds per plant (Clements et al., 2004), *Echinochloa crus-galli* up to 700,000 seeds (Holm et al., 1997), and *Portulaca oleracea* up to 1,800,000 seeds (Holm et al., 1997) Such high reproductive rates imply that even a few individuals can cause substantial weed populations in future growing seasons. Secondly, weeds possess sophisticated mechanisms for dispersing their seeds over large areas *via* wind, water, animals, and human activities (Umurzokov et al., 2024). Thirdly, many weeds exhibit seed dormancy (Zhang et al., 2019, 2023; Baskin & Baskin, 2014). This biological feature allows seeds to respond to varying environmental signals like temperature and lighting shifts, modulating dormancy to synchronize their growth initiation with the most optimal conditions and locales (Finch-Savage & Footitt, 2017). However, this biological feature also poses challenges in weed eradication, as dormant seeds can survive in the soil for extended durations (Zhang et al., 2022), thus escaping the impact of conventional weed control strategies.

Furthermore, in natural ecosystems, many weed seeds can form a dormancy cycle—a dynamic process in which seeds fluctuate between dormant and non-dormant states based on environmental cues (Baskin & Baskin, 2014). This cycle is crucial for the survival and propagation of weeds, particularly in regions with pronounced seasonal changes. It allows seeds to avoid germinating during brief periods of favorable conditions that do not last long enough to support full plant development (Zhang et al., 2023). Instead, seeds remain dormant until the onset of a prolonged favorable season, thereby optimizing their chances for successful germination and subsequent seedling survival (Baskin & Baskin, 2014). Moreover, climate change poses additional challenges for weed management by altering the environmental conditions that influence seed dormancy and germination (Reed, Bradford & Khanday, 2022; Zhang et al., 2024). As temperatures rise and precipitation patterns shift, the dormancy and germination cycles of weeds may change, potentially leading to more robust weed populations that are harder to control (Bernareggi et al., 2016; Maity et al., 2023).

*Diplachne fusca* (L.) P. Beauv. ex Roem. & Schult. (Poaceae), also known as bearded sprangletop, is a widely distributed grass species. It occurs as either an annual or short-lived perennial C4 grass and is distributed from Egypt to tropical Africa and South Africa, extending through Southeast Asia to Australia (Yuan et al., 2019; Driver, Al-Khatib & Godar, 2020a). Previous studies indicate that *D. fusca* has emerged as a significant issue in the coastal areas of Shanghai and Hebei provinces of China, becoming a dominant weed in certain paddy fields, thereby substantially diminishing both crop quality and yield (Yuan et al., 2019). Furthermore, this species is capable of developing a substantial seed bank, with up to 48,000 seeds per square meter found at depths of 0–6 cm (Morgan & Myers, 1989) and tolerates salty conditions (Myers & Morgan, 1989; Driver, Al-Khatib & Godar, 2020a). Additionally, *D. fusca* significantly reduces in agricultural production by competing crops for resources (Umurzokov et al., 2024), and climate change is expected to worsen its impact and diminish the effectiveness of chemical control (Rodenburg, Meinke & Johnson, 2011).

Currently, controlling *D. fusca* primarily involves herbicides. However, the extensive use of these chemicals may have prompted the evolution of herbicide resistance within weed species, with multiple instances of resistance to herbicides utilizing various mechanisms reported (Perotti et al., 2020; Montull & Torra, 2023; Ofosu et al., 2023). For example, Yuan et al. (2019) reported that *D. fusca* developed resistance to several herbicides such as metamifop, sethoxydim, and pinoxaden, with some populations exhibiting 8.9-fold resistance to cyhalofop-butyl compared to the susceptible population. Consequently, it becomes crucial to deepen our understanding of the biology and ecology of these plants to devise effective management strategies that do not rely solely on herbicides.

Germination of this species was studied by Morgan & Myers (1989), Myers & Morgan (1989), Hong et al. (1995), Altop et al. (2015), and Driver, Al-Khatib & Godar (2020a, 2020b). These studies found that high temperatures and light significantly enhance seed germination of this species (Morgan & Myers, 1989; Hong et al., 1995; Altop et al., 2015). Additionally, even under osmotic stress caused by −0.8 MPa of NaCl, approximately 50% of the seeds were still capable of germination (Myers & Morgan, 1989; Altop et al., 2015). Furthermore, seedling emergence decreased with increasing flooding and burial depths (Altop et al., 2015), and herbicide-resistant genotypes exhibited more seedling emergence than susceptible genotypes (Driver, Al-Khatib & Godar, 2020a, 2020b). Nevertheless, it remains unclear how the soil seed bank of *D. fusca* responds to environmental shifts, and whether a dormancy cycle is present in this species. Therefore, the primary aims of this study include: (1) identifying the specific requirements for dormancy loss and the kind of dormancy exhibited by this species; (2) determining the relationship between dormancy status and seasonal environmental shifts; (3) assessing the persistence of the soil seed bank; and (4) discussing the ecological adaptation strategies and potential methods for weed control based on the results obtained.

## Materials and Methods

### Seed collection

Freshly matured caryopses (hereinafter referred to as seeds) of *D. fusca* were gathered from numerous plants at Weifang University in Shandong Province, China (36.71°N, 119.18°E) at the beginning of September 2017. These seeds were air-dried at room temperature (18–24 °C with relative humidity 45–63%) for 1 week prior to starting germination experiments. All experiments were initiated within 2 weeks following the collection of the seeds. The collection site experiences a warm temperate monsoon climate, influenced by both terrestrial and marine elements, characterized by hot, wet summers and cold, dry winters. The yearly precipitation is 615.3 mm (Xiao et al., 2011), while the average annual temperature is 14.6 °C. January, the coldest month, experiences average temperatures of −0.48 °C. The peak temperatures occur in July with an average of 28.41 °C and an extreme maximum of 38 °C.

### General procedure for germination experiments

For all germination experiments, there were four replicates, each consisting of 25 seeds placed in a 5 cm diameter Petri dish lined with two layers of Whatman No. 1 filter paper moistened with 1 mL distilled water. The dishes were sealed with Parafilm to prevent moisture loss; additional water was added as needed during incubation to keep the filter paper moist. Germination was tested at 4 °C, 5/15 °C, 10/20 °C, 15/25 °C, and 20/30 °C. These temperatures were chosen to simulate local climate conditions: 4 °C for the cold of winter, 5/15 °C for early spring and late autumn, 10/20 °C for mid-spring, 15/25 °C for late spring and early autumn, and 20/30 °C for summer. Seeds were incubated under a 12-h light/dark cycle per day or in constant darkness (seeds placed in black bags). For those incubated under light conditions, the high and low temperatures corresponded to 12 h of daylight and 12 h of darkness, respectively, each day. Germination was defined as the radicle tip growing ≥1 mm. At the end of the experiment, seed viability was assessed according to Zhang et al. (2022).

### Germination of freshly matured *Diplachne fusca* seeds

Freshly matured seeds were incubated in both light and dark at 5/15 °C, 10/20 °C, 15/25 °C, and 20/30 °C for 14 days. This germination period was designed to be long enough to give the seeds ample time to germinate, while being short enough to prevent excessive warm (or cold) stratification that could break dormancy and thereby facilitate germination (Baskin & Baskin, 2014). For seeds incubated under light conditions, germination was monitored daily, and seeds that had germinated were discarded. In contrast, for seeds incubated under dark conditions, germination was monitored only at the end of the experiments.

### Effect of cold stratification on germination of *Diplachne fusca* seeds

To assess how cold stratification influences germination, a total of 1,000 fresh seeds were placed between two layers of filter paper, resting on a 3 cm-deep layer of sand with a moisture content of 11–14%, in four metallic containers (20 cm in diameter and 10 cm deep). The containers were sealed with lids and stored at a temperature of 4 °C for cold stratification. At intervals of 2, 4, and 8 weeks, four samples consisting of 25 seeds each were extracted from each container and placed into Petri dishes. These dishes were subsequently incubated under both light and dark 5/15 °C, 10/20 °C, 15/25 °C, and 20/30 °C for 14 days. Germination was monitored as described in “Germination of freshly matured Diplachne fusca seeds”.

### Effect of GA_3_ on germination of *Diplachne fusca* seeds

The influence of GA_3_ on germination is a key method used to identify dormancy kinds (Baskin & Baskin, 2014). Hence, fresh *Diplachne fusca* seeds were inoculated with 1 mL of GA_3_ solution at concentrations of 0 (water as the control), 0.01, 0.1, or 0.5 mmol/L. The Petri dishes were incubated in light at 20/30 °C for 14 days. Germination was monitored daily, and germinated seeds were discarded.

### Effect of dry storage (after-ripening) on germination of *Diplachne fusca* seeds

Freshly matured seeds of *D. fusca* were placed in open Petri dishes and subjected to after-ripening for periods of 2, 4, 8, and 12 weeks at room temperature (18–24 °C with relative humidity 45–63%). After each after-ripening period, the seeds were incubated in light at 20/30 °C for 14 days. Germination was monitored daily, and germinated seeds were discarded.

### Dormancy status of *Diplachne fusca* seeds buried in the field

To monitor seasonal variations in seed dormancy status, each of 480 nylon bags was filled with approximately 3,000 seeds and then buried at a soil depth of 2 cm within the experimental garden soil on campus, less than 2 km from the seed collection site. The bags were buried across two 4-square-meter plots, with the precise locations of each bag carefully documented. Starting on September 15, 2017, and on the first day of each month thereafter until October 2019, 10 bags were dug out from the soil and taken to the laboratory. In the lab, seeds and any seedlings were cleaned from the soil. Germinated seeds were removed, while non-germinated seeds were retained for further experiments. Each set of seeds was then incubated in both light and dark at 4 °C, 5/15 °C, 10/20 °C, 15/25 °C, and 20/30 °C. Germination was monitored as “Germination of freshly matured Diplachne fusca seeds”. At the end of the experiment, seed viability was assessed according to Zhang et al. (2022). The maximum and minimum air temperatures at the seed burial site were consistently monitored and recorded.

### Data analysis

Germination percentages were calculated based on the number of viable seeds. One-way ANOVA was conducted to investigate the effects of GA_3_ and the effects of the after-ripening period on the germination of *D. fusca* seeds. Furthermore, three-way ANOVAs were carried out to evaluate the impacts of cold stratification, temperature, and light, as well as the effects of the seed exhumation date, temperature, and light on the germination of these seeds. Significant differences identified by the ANOVAs were further examined using Tukey’s HSD *post-hoc* test (*p* < 0.05). All statistical analyses were performed using SPSS ver. 25.0.

## Results

### Germination of freshly matured *Diplachne fusca* seeds

**Figure 1:**
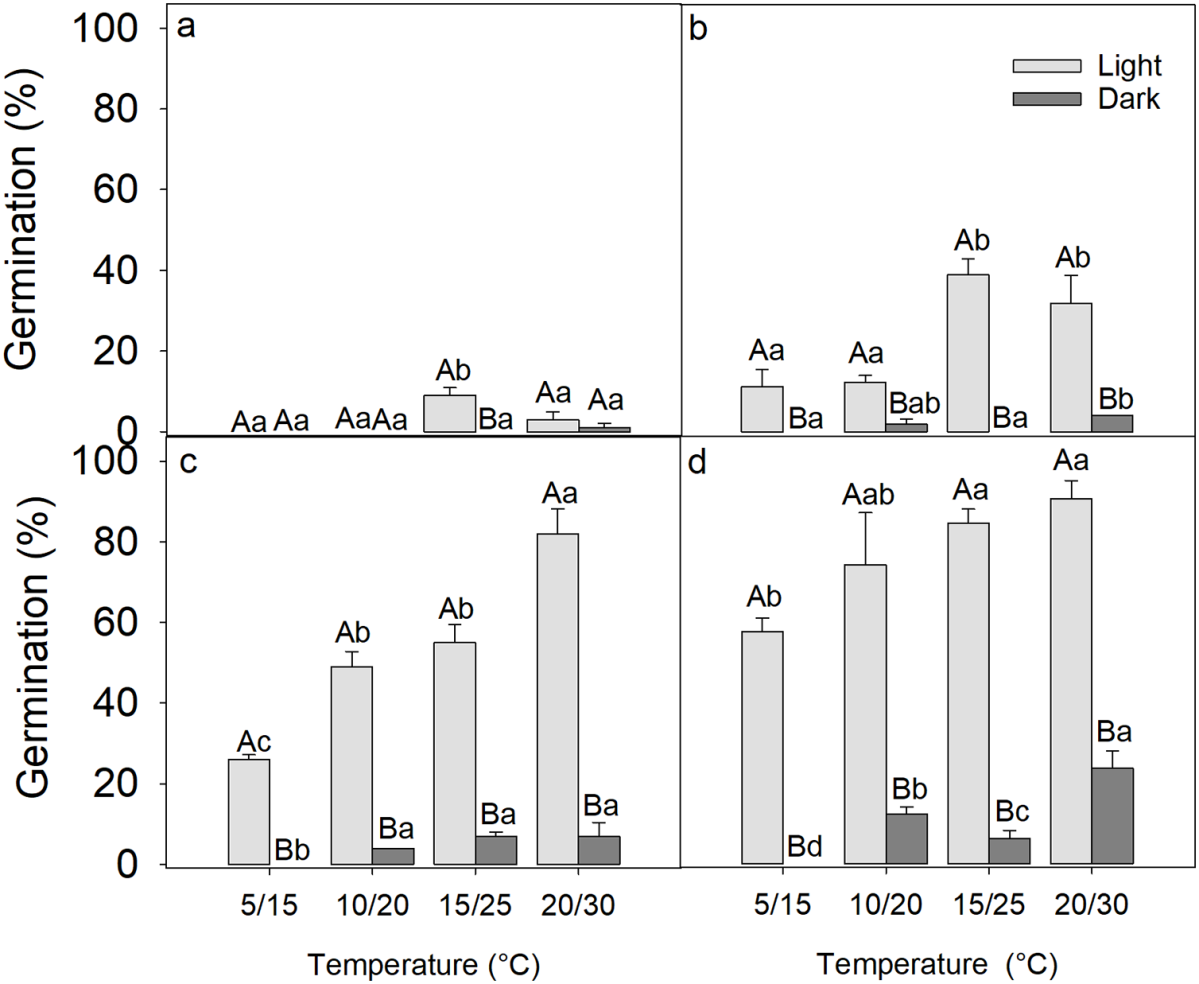
Influence of light exposure and 0 (A), 2 (B), 4 (C), 8 (D) weeks of cold stratification on the germination of *Diplachne fusca* seeds. Error bars represent mean ± SE. Different uppercase letters represent significant differences in germination within the same light condition across incubation temperatures, while different lowercase letters represent significant changes between light and dark conditions at the same incubation temperature (*p* < 0.05).

After 2 weeks of germination, the seed germination was only 9 ± 2% at 20/30 °C under light conditions, while in darkness, only one seed germinated at 20/30 °C, indicating that the freshly matured seeds of *D. fusca* were dormant (Fig. 1A).

### Effect of cold stratification on germination of *Diplachne fusca* seeds

A three-way ANOVA analysis revealed that the period of cold stratification, light exposure, incubation temperature, and all their two-way and three-way interactions significantly affected the germination of *D. fusca* seeds (Table 1). As the period of cold stratification was extended, there was a gradual improvement in seed germination percentages (Fig. 1). Specifically, following 0, 2, 4, and 8 weeks of cold stratification at 4 °C, the seeds reached 3 ± 1.9% (Fig. 1A), 31 ± 6.8% (Fig. 1B), 82 ± 6.2% (Fig. 1C), 88 ± 4.3% (Fig. 1D) germination, respectively, when incubated under light conditions at 20/30 °C. Conversely, germination in dark was only 1 ± 1.0% (Fig. 1A), 4 ± 0.0% (Fig. 1B), 7 ± 3.4% (Fig. 1C), and 23 ± 4.1% (Fig. 1D), respectively, indicating that darkness inhibited seed germination. Furthermore, the seeds showed significantly higher germination percentages at the higher incubation temperature (20/30 °C) compared to the lower temperature (5/15 °C) (Table 1).

**Table 1:** Full factorial three-way ANOVA analysis of the effect of stratification weeks, light, and temperature on germination of *Diplachne fusca* seeds.

Term	SS	df	MS	F value	*p* value
Stratification weeks (S)	6.854	3	2.285	192.680	<0.001
Temperature (T)	1.542	3	0.514	43.340	<0.001
Light (L)	7.548	1	7.548	636.571	<0.001
S × T	0.411	9	0.046	3.847	<0.001
S × L	2.322	3	0.774	65.281	<0.001
T × L	0.244	3	0.081	6.853	<0.001
S × T × L	0.323	9	0.036	3.023	<0.001

### Effect of GA_3_ and dry storage (after-ripening) on germination of *Diplachne fusca* seeds

**Figure 2:**
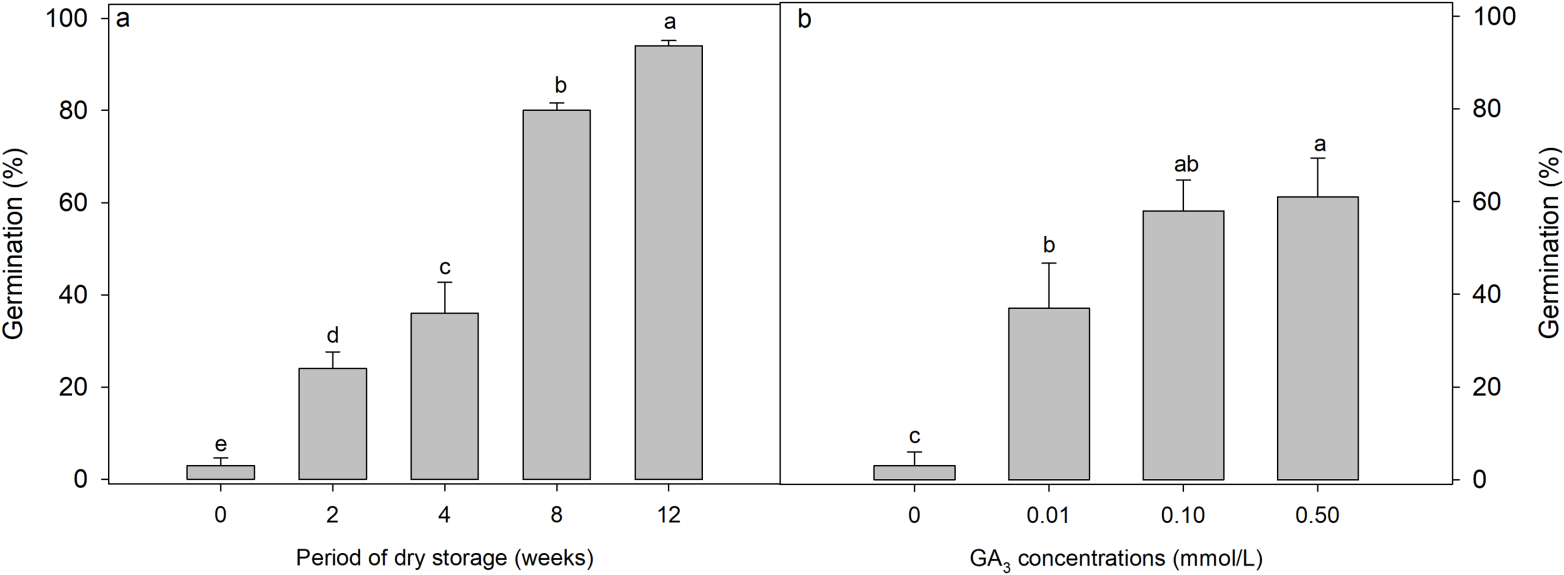
Influence of (A) dry storage (after-ripening) at room temperature (18–24 °C with relative humidity 45–63%) and (B) GA_3_ concentrations on the germination of Diplachne fusca seeds under light conditions at 20/30 °C. Different lowercase letters indicate statistically significant differences among treatments (storage weeks or GA_3_ concentrations) (*p* < 0.05).

Both GA_3_ concentration (F = 10.047, *p* < 0.001) (Fig. 2A) and after-ripening period (F = 127.746, *p* = 0.002) (Fig. 2B) significantly affected the germination of *D. fusca* seeds. Specifically, as the after-ripening period increased, germination of *D. fusca* seeds gradually increased (Fig. 2A). After 8 weeks of after-ripening, the germination reached 80 ± 1.6%. GA_3_ also enhanced seed germination, although not as effectively as after-ripening. Under GA_3_ concentrations of 0.1 and 0.5 mmol/L, seeds germinated to 58 ± 6.6% and 61 ± 8.4%, respectively (Fig. 2B).

### Dormancy status of *Diplachne fusca* seeds buried in the field

**Figure 3:**
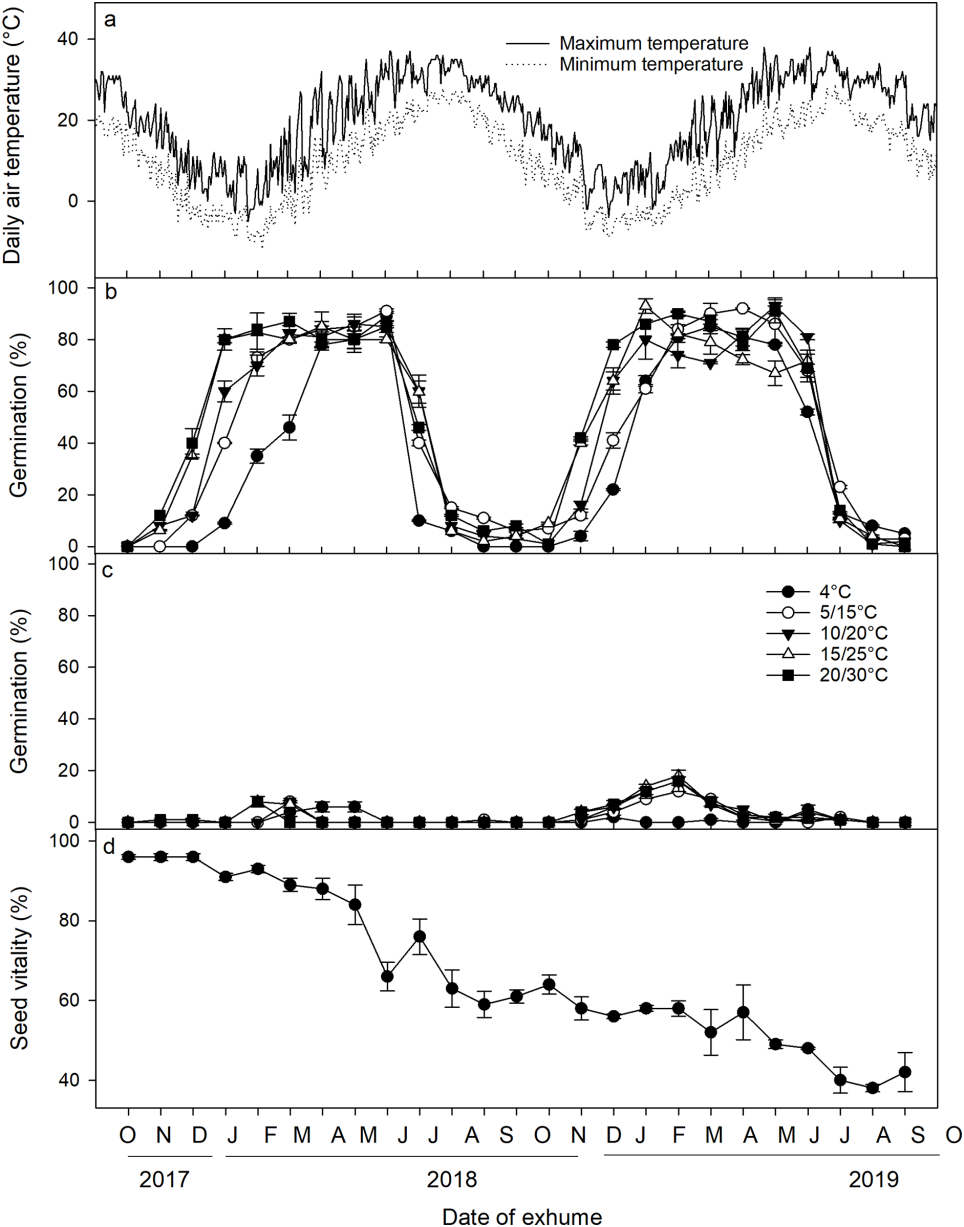
(A) Average daily maximum and minimum air temperatures; (B) germination percentages of *Diplachne fusca* seeds incubated under light; (C) and dark conditions at various temperature regimes; (D) and seed viability after 0–24 months of burial at 2 cm. Error bars represent mean ± SE.

A three-way ANOVA analysis revealed that the date of exhumation, incubation temperature, and light conditions, as well as their two-way and three-way interactions, had significant effects on seed germination (Table 2). Following excavation from the soil and subsequent incubation under laboratory conditions, a distinct cyclical pattern of germination percentages was observed (Fig. 3). Specifically, under light conditions, germination percentages began to increase in November 2017, reaching a peak in January 2018 when incubated at 5/15 °C, 10/20 °C, 15/25 °C, and 20/30 °C. Germination percentages at these temperatures remained high until June 2018 when the mean monthly maximum and minimum temperature in the field reached 31.7 °C and 20.5 °C, respectively, and thereafter, germination percentage sharply declined in July and August. From September to November, only a minimal number of seeds germinated. Germination percentages then increased once more from November 2018 to February 2019, only to decrease once more in July 2019. Under dark conditions, <20% of the seeds germinated across the year. Seed viability showed a gradual decrease (F = 44.082, *p* < 0.001), particularly notable from May to August. Nevertheless, even after 2 years of burial, ca. 40% of the seeds retained their viability (Fig. 3D).

**Table 2:** Full factorial three-way ANOVA analysis of the effect of date of exhume, light, and temperature on *Diplachne fusca* seed germination.

Term	SS	df	MS	F value	*p* value
Date of exhume (Date)	53.558	24	2.232	836.299	<0.001
Light (L)	84.773	1	84.773	31769.479	<0.001
Temperature (T)	2.629	4	0.657	246.264	<0.001
Date × L	33.577	24	1.399	524.309	<0.001
Date × T	4.774	96	0.05	18.635	<0.001
L × T	0.661	4	0.165	61.916	<0.001
Date × L × T	3.084	96	0.032	12.039	<0.001

## Discussion

Our results on *Diplachne fusca* seeds align with those from previous studies that found these seeds exhibit dormancy (Morgan & Myers, 1989; Myers & Morgan, 1989; Hong et al., 1995; Driver, Al-Khatib & Godar, 2020a). Similar to those studies, we observed that after-ripening at room conditions can enhance seed germination, and seeds exhibited a higher germination percentage in light than in darkness. However, in contrast to Morgan & Myers (1989) but consistent with Hong et al. (1995), we found that cold stratification could promote the breaking of dormancy (Fig. 1). This variance may stem from the different geographic origins of the seeds: those in the study by Morgan & Myers (1989) were collected from southeastern Australia, while the seeds in the studies by Hong et al. (1995) and our study were sourced from East Asia. The geographical variation in seed dormancy response suggests that local environmental conditions may significantly influence the physiological traits of seeds, impacting adaptability and survival strategies of plant species across different climates and habitats (Baskin & Baskin, 2014).

According to Baskin & Baskin (2014), seeds within the Poaceae family may exhibit physiological dormancy (PD) or none at all. PD is divided into three levels: non-deep, intermediate and deep (Baskin & Baskin, 2014). Non-deep PD can be overcome by brief warm or cold stratification, gibberellic acid (GA_3_), or after-ripening (Soltani, Baskin & Baskin, 2017). Intermediate PD generally requires a longer period of cold stratification, and GA_3_ works effectively only in certain species. Deep PD, on the other hand, can only be alleviated by prolonged cold stratification. Given that GA_3_ and after-ripening can enhance seed germination, and that 8 weeks of cold stratification results in 80% seed germination, we conclude that the seeds of *Diplachne fusca* exhibit non-deep PD.

Dark inhibited germination of *D. fusca* seeds (Fig. 1). It seems to be a common phenomenon in weed seeds. For example, darkness suppresses the germination percentages of seeds from *Bidens pilosa* (Chauhan, Ali & Florentine, 2019), *Hibiscus tridactylites* (Chauhan, 2016), *Lolium rigidum* (Thompson, Mahajan & Chauhan, 2021), and *Nassella trichotoma* (Humphries, Chauhan & Florentine, 2018). The ecological explanation of this phenomenon is that light serves as a signal of soil depth, preventing germination when seeds are buried too deep (Chauhan, Ali & Florentine, 2019). Since weed seeds are typically small, this depth-sensing mechanism helps avoid the potentially fatal germination of seeds that are too deeply buried for seedlings to grow enough to reach the soil surface (Chauhan, Ali & Florentine, 2019). During tillage, seeds may be moved to the surface by the plow, enabling them to germinate quickly (Schutz, Milberg & Lamont, 2002).

While light requirements usually prevent non-dormant seeds from germinating when buried in soil, whether such seeds will immediately germinate after being placed on a moist, well-lit soil surface depends on their physiological state and ambient temperatures. The dormancy of buried seeds of *D. fusca* was broken during the winter, and these seeds had a high germination capability in January, even although the mean maximum temperature was only 3.19 °C. They were still able to germinate under laboratory conditions, indicating that their dormancy had been broken. A similar requirement for dormancy breaking and early germination has also been observed in summer annuals like *Bidens pilosa* (Zhang et al., 2019), *B. polylepis* (Baskin, Baskin & Chester, 1995), *Eleocharis palustris* (Rosbakh et al., 2019), and *Ambrosia artemisiifolia* (Baskin & Baskin, 1980). Such early dormancy breakage allows seeds to germinate early in the growing season and thus maximize plant growth, flowering, and seed production, and this germination strategy also reduces competition with other species.

After dormancy was broken, the seeds were capable of germinating until early summer (June), when mean monthly maximum and minimum field temperatures reached 31.7 °C and 20.5 °C, respectively (Fig. 3A). It is important to note that although we observed that the seeds began to lose their ability to germinate in July, this loss of germination capability might not necessarily be due to the high temperatures of July. It is possible that the induction of secondary dormancy in the seeds could have started earlier. For instance, after four weeks of incubation at 15/25 °C, the non-dormant seeds of *Amaranthus palmeri* (Jha et al., 2010) and *Ambrosia artemisiifolia* (Baskin & Baskin, 1980) lost their germination capability when transferred to different temperatures. However, in the case of *Bidens polylepis* (Baskin, Baskin & Chester, 1995), non-dormant seeds required 12 weeks of warm temperatures before they lost their ability to germinate. To determine what temperatures and duration of exposure cause the non-dormant seeds of *D. fusca* to enter secondary dormancy, further laboratory work is needed. Nevertheless, our study indicates that seeds of *D. fusca* exhibit secondary dormancy and a clear dormancy cycle.

After 2 years of burial, approximately 40% of the seeds remained viable. Thus, seeds of *D. fusca* can form at least a short-lived persistent seed bank. Previous studies have shown that soil seed banks are essential for sustaining populations in environments that experience fluctuations (Gioria et al., 2020; DeMalach, Kigel & Sternberg, 2021; Plue et al., 2021). For example, seeds of *Ambrosia artemisiifolia* (Asteraceae) were still able to germinate after 40 years of burial in the soil (Baskin & Baskin, 1977). Therefore, persistent and long-term control measures are essential to effectively manage this weed due to its ability to maintain resilience in seed banks.

From an ecological and evolutionary perspective, the secondary dormancy of *D. fusca* seeds during summer ensures that the seeds do not germinate in late summer or fall, a time when they could not complete their life cycle or when autumn-germinated seedlings might succumb to freezing temperatures. Moreover, the requirement for light to trigger germination plays a significant role in the life cycle of *D. fusca*. Light availability is typically a sign of an open space in vegetation—areas where competition for water, nutrients, and space is reduced (Eskelinen et al., 2022). By germinating in response to light, *D. fusca* seeds can quickly capitalize on these openings, taking root and establishing themselves before other species have a chance to compete for the same resources. This ability is especially beneficial following disturbances that clear vegetation, such as fire or human activities, which create immediate opportunities for colonization. Furthermore, a persistent seed bank plays a crucial role in the plant’s survival under adverse conditions such as drought, fire, or unfavorable seasonal climates. Seeds that remain viable in the soil have the potential to germinate when conditions eventually become favorable, thus ensuring species resilience through unpredictable environmental changes. All these factors collectively contribute to the successful reproductive strategy of *D. fusca* at the seed stage.

Nevertheless, from an agricultural perspective, the characteristics of *D. fusca* seeds pose significant challenges for crop management and farming practices. The need for light to germinate means that typical soil disturbance activities, such as plowing or tilling, which are intended to prepare the land for crops, can inadvertently bring seeds to the surface and trigger a mass germination of weed seeds (Chauhan, Ali & Florentine, 2019). The secondary dormancy and persistence in soil indicate that these seeds can survive in the soil for many years. To effectively manage *D. fusca* in agriculture, we recommend the following strategies. First, employing deep plowing techniques can bury seeds beyond the reach of light necessary for germination and would reduce the likelihood of weed emergence. Second, integrating the use of cover crops can effectively suppress the growth of *D. fusca* by competing for light, nutrients, and space, and would prevent weed seeds from finding the conducive conditions they need to sprout. Third, adjusting the timing of tillage to avoid soil disturbances during peak germination periods can prevent bringing nondormant seeds to the surface where they can germinate. By implementing these targeted management strategies, farmers can reduce the impact of *D. fusca* and support sustainable farming practices by avoiding the development of herbicide-resistant weed populations, which can arise from excessive reliance on chemical controls (Fanadzo, Dalicuba & Dube, 2018).

Our study clearly has limitations. Firstly, *D. fusca* is a widely distributed species, and its habitat ranges from roadside to paddy fields as well as intermittent wetlands. We only collected the seeds from the campus; however, different habitats might select for different phenotypes (adaptations) (Radersma, Noble & Uller, 2020; Wadgymar et al., 2022). Secondly, we only applied a single burial depth (2 cm). Seeds buried at different depths can experience varying levels of moisture, temperature, and oxygen availability, all of which can significantly affect germination (Amini, Gholami & Ghanepour, 2017; Tao et al., 2022; Sousa-Ortega et al., 2023). Furthermore, we did not study the effect of flooding. Given that the seeds we collected were from a non-flooding habitat, will populations from flooding habitats and non-flooded habitats respond differently to flooding? Will the timing and duration of flooding events influence the effectiveness of dormancy break? Will the physiological adaptations to flooding conditions vary between different habitats? Future studies considering these factors would provide more applicable management strategies for controlling *D. fusca* in various habitats.

## Conclusions

Our study indicates that the newly ripened seeds of *Diplachne fusca* are dormant, with germination facilitated by GA_3_, cold stratification, and after-ripening at ambient room conditions, while darkness inhibits germination. Seeds that do not germinate in spring and early summer enter secondary dormancy, and approximately 40% of seeds remain viable after 2 years of burial, indicating a persistent seed bank. From an ecological perspective, secondary dormancy prevents late summer or fall germination, which prevents seedlings from begin killed by freezing temperatures, while light-triggered germination allows seeds to capitalize on open spaces. These traits present significant challenges for agricultural management, as typical soil disturbances can bring seeds to the surface and trigger mass germination. Effective management strategies include deep plowing, cover crops, and adjusting tillage timing to avoid peak germination periods. Future studies investigating the impact of different habitats, burial depths, and flooding conditions on *D. fusca* dormancy and germination would provide more comprehensive management strategies for this weed species.

## Supplemental Information

10.7717/peerj.17987/supp-1 Supplemental Information 1 Seed germination raw data. Each data point indicates the percentage of seeds germinated.

## Additional Information and Declarations

### Data Availability

The following information was supplied regarding data availability: The raw data are available in the Supplemental File.
